# Supplementary material for: Experiences, distress and burden among neurologists in Norway during the COVID-19 pandemic
Source: PLoS One. 2021 Feb 4;16(2):e0246567. doi: 10.1371/journal.pone.0246567 (PMC7861439; doi:10.1371/journal.pone.0246567)
Supplement: S1 File — (PDF) [file pone.0246567.s001.pdf]

Neurology during a pandemic (Norway, April 2020)

This questionnaire represents an effort to map experiences and management within neurology during the COVID-19 pandemic in Norway. Doctors who work clinically within neurology in Norway are invited to fill in this questionnaire. The questionnaire will not identify individual departments or hospitals. All questions are scored in relation to the ongoing pandemic. Study-related questions can be directed to the project manager (ESK).

Your age?

Gender (female/male)?

Resident or senior consultant in neurology?

Do you work at a University Hospital? (yes/no)

What is your work situation like now as compared to normal? (yes/no/not applicable)
Changed
My duties are unchanged, but I work more than before
My duties are changed, and I work more than before
My duties are unchanged, but I work less than before
I have been relocated from neurology to another ward
Our work schedule has changed
We have extended the doctors` working hours
If you have a research position, has research time been revoked?
I am satisfied with the work
We have reduced the number of beds
We have maintained the regular out-patient clinic (with in-person appointments as the norm)
We have switched to primarily telephone/video consultations
We assess newly referred patients via telephone consultation
We assess newly referred patients via video consultation
We do follow-ups via telephone consultation
We do follow-ups via video consultation
Telephone/video consultations are more effective and better than the regular in-person appointment
Do you spend less time on telephone/video consultations than at the regular in-person appointment
From a professional perspective, are telephone consultations with newly referred patients satisfactorily
From a professional perspective, are video consultations with newly referred patients satisfactorily
From a professional perspective, do follow-ups by telephone consultation work satisfactorily
From a professional perspective, do follow-ups by video consultation work satisfactorily
We have been able to facilitate home office

Any other comments related to work situation? ___________________________

Please rate the following statements (Strongly agree/agree/ neither agree nor disagree/disagree/strongly disagree)
I experienced that the academic community in Norway collaborated to find good solutions for neurological patients
I experienced that the health authorities in Norway collaborated to find good solutions for neurological patients
Fewer patients have been referred to the emergency ward with potentially neurological conditions
Fewer patients have been admitted from the emergency ward to the neurology department with potentially neurological conditions

Reduced standard of care is available for patients with acute neurological conditions
Patients with acute neurological conditions had a worse prognosis
Patients who would normally be admitted to the neurology department for acute treatment are being sent home without admission
Patients who would normally be admitted to the neurology department for further sub-acute investigations are being sent home without admission (investigations postponed)
In-patient elective treatments are postponed
In-patient elective investigations are postponed
Reduced standard of care is available for patients with chronic neurological conditions
Patients with chronic neurological conditions had a worse prognosis
Patients have come for treatment and admissions later than usual (patient delay)
Hospitalised patients are considerably sicker now than before

To what degree are you stressed or burdened by the following situations/factors related to the COVID-19 pandemic, 1=No burden and 5=Very significant burden.
My job impacts negatively on my family life
Finding balance between my work and private life
My job impacts negatively on my social life
The fear of contracting SARS CoV-2 at work impacts negatively on my work
The fear of contracting SARS CoV-2 at work impacts negatively on my quality of life
I am afraid that I will spread SARS CoV-2 to close family members
Potential lack of personal protection equipment in my clinical work
Patients are not given the follow-up they should receive
Changed work routines and access to resources

At any point, have you felt down or depressed for 2 weeks in the last 4 weeks due to your job as a neurologist? (yes/no)
My sleep has suffered due to the pandemic? (yes/no)
Have you assessed patients with clinical suspicion of COVID-19? (yes/no)
Approximately how many patients with clinical suspicion of COVID-19 have you assessed? (<10, 10-30, 31-50, >50)

The following questions relate to the management of some of the largest disease groups within neurology. When you answer “yes” to a disease group, a few more questions specifically connected to that disease will come up. Only answer for the patient groups that you have some experience with.

I assess patients with acute stoke (yes/no/not applicable)
Has the pandemic affected your course of treatment for stroke in your department
Fewer patients are receiving thrombolysis than before the pandemic
We have changed the thrombolysis procedures (practical implementation)
We spend more time than usual on thrombolysis assessments
We have increased the proportion of patients who receive MRI in the acute phase
We have increased the proportion of patients who receive CT angiography in the acute phase to avoid carotid ultrasound
We have reduced the proportion of patients who receive carotid ultrasound to avoid SARS-CoV2 exposure
We are still admitting TIA patients
Stroke patients have reduced access to rehabilitation
We discharge patients faster than usual
Fewer stroke patients are coming to the hospital than usual
The stroke patients who come to the hospital are more severely affected than those we usually see

Stroke patients arrive at the hospital later than usual
Does the hospital where you work usually perform thrombectomy
If yes, have changes been made to the thrombectomy procedures
If yes, are fewer patients offered thrombectomy
If yes, are patients offered thrombectomy even though they have unknown infection status and have to be placed under general anesthesia during the procedures
Fewer patients receive extended etiologic assessment (telemetry, Holter, ultrasound)
Out-patient follow-ups after stroke are postponed
Reduced standard of care has been available for stroke patients during the pandemic

I assess headache patients at in out-patient clinic (yes/no/not applicable)
Have you seen newly referred headache patients during the pandemic
If you assess newly referred patients by telephone consultation, does it work satisfactorily for the patients
If you assess newly referred patients by telephone consultation, does it work satisfactorily for you
If you are conducting follow-up with headache patients by telephone consultation, does it work satisfactorily for the patients
If you are conducting follow-up with headache patients by telephone consultation, does it work satisfactorily for you
Have you been more likely to put patients on CGRP antibodies rather than botulinum toxin
Do you refrain from using CGRP antibodies
Have you switched more patient than usual from botulinum toxin to CGRP antibodies
Headache patients receive the same follow-up as usual
Reduced standard of care has been available to headache patients during the pandemic

Do you continue with botulinum toxin treatment for chronic migraine during the pandemic
- Yes, with the usual 12-week intervals
- Yes, but with delays, and therefore increased intervals
- Treatment is limited to a few selected patients
- No, as a rule we administer no botulinum toxin treatment
- We do not start any new patients on botulinum toxin treatment
- We did not offer botulinum toxin treatment before the pandemic

I treat patients with epilepsy (admission/ward or out-patient clinic) (yes/no/not applicable)
We have maintained the regular epilepsy care
Acute treatment for admitted patients with seizures is changed
Status epilepticus treatment is worse than usual
As many patients as usual are sedated and intubated as part of status epilepticus treatment
There is worse access to emergency EEG
Out-patient follow-ups are postponed
Out-patient follow-up are primarily conducted by telephone consultation
Out-patient follow-up are primarily conducted by video consultations
If you have started conducting telephone/video consultations for epilepsy patients during the pandemic, do they function satisfactorily for the patients
If you have started conducting telephone/video consultations for epilepsy patients during the pandemic, do they function satisfactorily for you
From a professional perspective, is it satisfactory to make dosage changes for epilepsy patients by the telephone
From a professional perspective, is it satisfactory to assess frequency and type of seizure by the telephone
Access to elective EEG and sleep-deprived EEG is worse
We take fewer medication analysis
Driving licence follow-ups are postponed
Reduced standard of care has been available to epilepsy patients during the pandemic

I treat patients with multiple sclerosis (ward/out-patient clinic) (yes/no/not applicable)
We have maintained the regular multiple sclerosis care
Treatment of acute attacks is less available
I have asked patients to stay away from the hospital because they are a vulnerable group
Out-patient follow-ups are postponed
Out-patient follow-up are primarily conducted by telephone consultation
Out-patient follow-up are primarily conducted by video consultation
If you have started conducting telephone/video consultations for multiple sclerosis patients during the pandemic, do they function satisfactorily for the patients
If you have started conducting telephone/video consultations for multiple sclerosis patients during the pandemic, do they function satisfactorily for you
From a professional perspective, is it satisfactory to assess newly referred multiple sclerosis patients by telephone consultations
I spend less time on telephone consultations with multiple sclerosis patients than at the usual in-person appointment
We have chosen different types of treatment for newly diagnosed patients than we normally do
We have changed the current treatment for individual patients due to the pandemic
Overall, I think the prognosis for MS patients as a group is worse during the pandemic
I have read the recommendations from the National Advisory Unit on multiple sclerosis and Covid-19
Reduced standard of care has been available to multiple sclerosis patients during the pandemic

I treat patients with movement disorders (out-patient clinic) (yes/no/not applicable)
We have maintained the regular movement disorders care
Out-patient follow-ups are postponed
Out-patient follow-up are primarily conducted by telephone consultation
Out-patient follow-up are primarily conducted by video consultation
If you have started conducting telephone consultations for movement disorder patients during the pandemic, do they function satisfactorily for the patients
If you have started conducting telephone consultations for movement disorder patients during the pandemic, do they function satisfactorily for you
Are you more likely to postpone altering dosages after a telephone consultation than a face-to-face meeting
Some newly referred movement disorder patients are assessed by telephone consultations
From a professional perspective, is it satisfactory to assess newly referred movement disorder patients by telephone consultations
I have patients whose advanced Parkinson`s treatment has been postponed as a consequence of the pandemic
Reduced standard of care has been available to movement disorder patients during the pandemic

I assess patients with amyotrophic lateral sclerosis (yes/no/not applicable)
We have maintained regular ALS out-patient clinic
I have recommended that ALS patients avoid hospital because they should not be exposed to potential Covid-19 infection at the hospital
We offer telephone consultations to ALS patients
I have patients whose home respirator has been delayed
I have patients who has been denied home respirator
Patients with ALS have a decreased lifespan due to worse options from the health services during the pandemic
Reduced standard of care has been available to amyotrophic lateral sclerosis patients during the pandemic

I assess patients with glioblastoma (yes/no/not applicable)
Out-patient follow-ups are postponed
Out-patient follow-ups are postponed for administrative reasons

Out-patient follow-ups are postponed because these patients should not be exposed to potential Covid-19 infection at the hospital
Treatment with temozolomide is given as planned
Diagnostic examination of glioblastoma patients takes longer time
There is longer waiting time before surgery for newly diagnosed glioblastoma patients
Radiation therapy are postponed
We avoid admitting patients with glioblastoma even when their condition is worsening because they should not be exposed to potential Covid-19 infection at the hospital
Patients with glioblastoma have a decreased lifespan due to worse options from the health services during the pandemic
Reduced standard of care has been available to glioblastoma patients during the pandemic

Do you assess patients who are being treated with immunoglobulins or other immunological treatments for various polyneuropathies (yes/no/not applicable)
Patients receive immunological treatment with regular intervals
We extend the intervals
Immunological treatment of new patients begins as planned
We have postponed all treatment until we know more about Covid-19 and this type of treatment
Reduced standard of care has been available to patients with immune-mediated polyneuropathies during the pandemic

Do you treat patients with dystonia with botulinum toxin (yes/no/not applicable)
We continue to offer botulinum toxin at regular intervals
If not, do you believe that this has a negative impact on the patients´ function level and quality of life in the long term
Reduced standard of care has been available to patients with dystonia during the pandemic

Do you treat patients with spasticity with botulinum toxin (yes/no/not applicable)
We continue to offer botulinum toxin at regular intervals
If not, do you believe that this has a negative impact on the patients´ function level and quality of life in the long term
Reduced standard of care has been available to patients with spasticity during the pandemic

Overall, which group of neurology patients do you feel suffer most due to changes that may have been made at your department during the pandemic_____________________

Thank you for your assistance!
