# Supplementary material for: Experiences, distress and burden among neurologists in Norway during the COVID-19 pandemic
Source: PLoS One. 2021 Feb 4;16(2):e0246567. doi: 10.1371/journal.pone.0246567 (PMC7861439; doi:10.1371/journal.pone.0246567)
Supplement: Attachment — Submitted filename: Responses to reviewers.docx [file pone.0246567.s002.docx]

**Additional Editor Comments:**

Dear Prof Saxhaug Kristoffersen
Your study is well-crafted and received favourable opinions from Reviewers.

Thank you for the kind words.

Please fulfill their suggestions in order to further improve your paper quality

Journal Requirements:

When submitting your revision, we need you to address these additional requirements.

1. Please ensure that your manuscript meets PLOS ONE's style requirements, including those for file naming. The PLOS ONE style templates can be found at

https://journals.plos.org/plosone/s/file?id=wjVg/PLOSOne_formatting_sample_main_body.pdf and

<https://journals.plos.org/plosone/s/file?id=ba62/PLOSOne_formatting_sample_title_authors_affiliations.pdf>

We have changed the manuscript and files accordingly.

2. In line with PLOS' guidelines on replicability and reporting (https://journals.plos.org/plosone/s/criteria-for-publication#loc-3), please include additional information regarding the survey or questionnaire used in the study and ensure that you have provided sufficient details that others could replicate the analyses. For instance, if you developed a questionnaire as part of this study and it is not under a copyright more restrictive than CC-BY, please include a copy, in both the original language and English, as Supporting Information.

We have added the questionnaire as supporting information.

3. Thank you for stating the following in the Competing Interests section:

"BWS has received speaking fees from Novartis, unrelated to the present work. ECS has received speaking fees from Bayer and Novartis, unrelated to the present work. AMS, ESK, KWF and SHJ report no conflicts of interest."

Please confirm that this does not alter your adherence to all PLOS ONE policies on sharing data and materials, by including the following statement: "This does not alter our adherence to PLOS ONE policies on sharing data and materials.” (as detailed online in our guide for authors [http://journals.plos.org/plosone/s/competing-interests](about:blank)).  If there are restrictions on sharing of data and/or materials, please state these. Please note that we cannot proceed with consideration of your article until this information has been declared.

Please include your updated Competing Interests statement in your cover letter; we will change the online submission form on your behalf.

Please know it is PLOS ONE policy for corresponding authors to declare, on behalf of all authors, all potential competing interests for the purposes of transparency. PLOS defines a competing interest as anything that interferes with, or could reasonably be perceived as interfering with, the full and objective presentation, peer review, editorial decision-making, or publication of research or non-research articles submitted to one of the journals. Competing interests can be financial or non-financial, professional, or personal. Competing interests can arise in relationship to an organization or another person. Please follow this link to our website for more details on competing interests: [http://journals.plos.org/plosone/s/competing-interests](about:blank)

We have added an updated Competing Interests statement in the cover letter.

"BWS has received speaking fees from Novartis, unrelated to the present work. ECS has received speaking fees from Bayer and Novartis, unrelated to the present work. AMS, ESK, KWF and SHJ report no conflicts of interest. This does not alter our adherence to PLOS ONE policies on sharing data and materials.”

**Reviewers' comments:
Comments to the Author**

Reviewer #1: This very informative and well written analysis (manuscript ID of PONE-D-20-40374) reports experiences, distress and burden among Norwegian neurologists during the first weeks of the COVID-19 pandemic based on a web-based survey. The authors report changes in the personal work situation for the vast majority of neurologists in Norway with a high degree of burden and distress during the early pandemic, which was mainly driven by changed access to resources, fearing lack of personal protection equipment and spreading the virus to family members rather than becoming ill. This work is an important contribution to understand the burden of the pandemic among physicians. The authors identify problems and fears of Norwegian neurologists that could be helpful for this still ongoing pandemic. I have only minor concerns regarding this manuscript.

Thank you for these nice comments.

My detailed comments and questions are as follows:

Methods:
This descriptive analysis is well demonstrated. The data is fully available.
Am I right that the online questionnaire was send out and received again in April 2020? How much time did the participates have to consent to the study an answer the questionnaire? Recall bias is the main limitation, especially if the time window for answering this questionnaire was too small in this busy phase of the early pandemic.

Yes, you are right. The online questionnaire was sent out in April 2020 and the study was concluded in May. The participants had approximately three weeks to answer the questionnaire. We have added information about this in the methods.

Results/Discussion:
Was there an association between depressive thoughts and the factors that caused burden and stress to the neurologists, especially for those factors that caused a high degree of burden (changed access to resources, fearing lack of personal protection and spreading the virus to family members)?
Thank you for this great suggestion. We have added these additional analyses in the result section.

It would be interesting to know the authors´ opinion about how to tackle some of the problems that caused stress during the early pandemic. Thus, it might be possible to reduce the burden of the still ongoing pandemic. For example, what do the authors think about screening health care workers for depressive symptoms in such an exceptional situation like a pandemic?

We agree with the reviewer that this is interesting. Some of the stress is probably unavoidable, but we have elaborated on factors that may be possible to control and impact. First of all, we think that access to adequate PPE, but also realistic plans on how to strategically use the workforce including work schedules, academic and social support, and reasonable time to recover must be in place to avoid this increased burden placed on many health care workers. For the second part of the question – it is probably too early to know to what degree the pandemic will have long-term impact on the mental health of health care workers, but that is by no means unlikely. The main problem with screening of health care workers will probably be how to handle the answers when the total health care system is on the brink of collapse.

Reduced standard of care for stroke was reported by 48% of the participants and 83% reported changes in the thrombolysis procedures. This is very interesting. Are there data available concerning the percentage of treatment with thrombolysis in AIS patients in the early phase of the pandemic in Norway? For example, other countries recently reported a decrease in the absolute thrombolysis and thrombectomy treatment numbers but similar treatment rates compared with previous months for those AIS patients that presented to hospital (<https://doi.org/10.1161/STROKEAHA.120.033160>).

Yes, we have very recently published two papers on stroke admission rates in Norway before, during and after the initial phase of the pandemic. In Norway, as in Germany, we found a decrease in the absolute thrombolysis treatment numbers, but similar treatment rates compared with previous months for those with ischemic stroke that presented to hospital within the 4.5 hour time window. We have added more on this (including references to the German and Norwegian studies) in the discussion.

Reviewer #2: I read with much interest this paper. In general terms it is well written and clearly organised. I have just a few minor comments:

Thank you for these nice comments.

- I would recommend to specify exact time frame and place in the context of the pandemic wave in Norway providing details on the situation at that time (ie lockdown other measures). This because in 10 years time some people may not recollect many details.

We have added this information in the methods, under settings.

- I would recommend citing other studies who have investigated the same issues in other countries or in other medical specialties and to discuss differences

Thank you for this suggestion. We have elaborated on this in the discussion.

- I would recommend to specify in the title that the paper focuses on neurologists in Norway

We have added “in Norway” in the title. The title is now “Experiences, distress and burden among neurologists in Norway during the COVID-19 pandemic”
